# Latent profile analysis of psychological help-seeking stigma and influential factors among Hainan medical students

**DOI:** 10.1371/journal.pone.0319680

**Published:** 2025-04-16

**Authors:** Yingqi Li, Jiangyou Long, Shuting Huang, Hongbing Xu, Kaixin Wangzhou, Lei Qiu

**Affiliations:** 1 School of Public Health, Hainan Medical University, Hainan, People’s Republic of China; 2 Department of Social Medicine and Health Management, School of Public Health, Tongji Medical College, Huazhong University of Science and Technology, Wuhan, People’s Republic of China; 3 School of Management, Hainan Medical University, Hainan, People’s Republic of China; University of Chinese Academy of Sciences, CHINA

## Abstract

**Objective:**

Medical students frequently confront a range of psychological challenges inherent to their profession. Among these, the psychological help-seeking stigma (PHSS) is a crucial yet often neglected barrier, impeding medical students from obtaining necessary psychological support. This research aims to elucidate the characteristics of PHSS in medical students through latent profile analysis (LPA), examine its psycho-social determinants, and establish the threshold for PHSS using receiver operating characteristic (ROC) analysis.

**Methods:**

A cross-sectional survey was conducted among 3650 medical students from three medical colleges in Hainan Province, China, between February and July 2023. The survey gathered data on demographic details, PHSS, family socioeconomic status, professional help-seeking attitude, mental health level, self-efficacy and family function. LPA was employed to categorize distinct stigma profiles. Univariate and multiple logistic regression analyses were utilized to investigate the factors influencing these PHSS profiles. ROC analysis was executed to determine the optimal cut-off value for PHSS.

**Results:**

Three distinct PHSS profiles emerged among the participants: “low-level stigma” (23.2%), “moderate-level stigma” (53.2%), and “high-level stigma” (23.6%). Logistic regression analysis revealed that professional help-seeking attitude, mental health level, self-efficacy and family function were associated with PHSS. The ROC analysis determined that the optimal cut-off value for identifying PHSS was ≥  19.5.

**Conclusion:**

This study highlights the substantial variability in PHSS among medical students. The identification of key influencing factors underscores the need for bespoke mental health interventions tailored to this unique demographic.

## 1. Introduction

With the progress of society and the deepening of people’s understanding of mental health, more and more institutions and schools have set up psychological counseling rooms to provide necessary support for individuals [1]. Although these resources have been provided, their utilization rate is relatively low, especially in higher education institutions [2]. Because of their professional characteristics and academic pressure, medical students face more unique and complex psychological challenges, so their mental health is particularly noteworthy [3]. However, due to the psychological help-seeking stigma, many medical students rarely take the initiative to seek psychological help [4,5].

Psychological help-seeking stigma (PHSS) is an individual’s negative attitude or cognition towards seeking psychological help, which may cause medical students not to seek psychological help for fear of being discriminated against by peers or society [6]. In addition, PHSS may aggravate the pressure of medical students in clinical practice, interpersonal communication and future career development, further amplify their mental health risk, and lead to the occurrence of depression, anxiety, self-injury and other problems [3].

Despite some research conducted by scholars on PHSS among medical students, there are evident shortcomings in the existing body of research. These studies predominantly employ traditional statistical methods, such as descriptive statistics and correlation analysis, which are insufficient to deeply elucidate the heterogeneity and complexity of PHSS among medical students. Moreover, the research is often confined to unidimensionalperspective, lacking identification and discussion of potential categories of PHSS. This limitation limits our comprehensive understanding of the PHSS spectrum among medical students and fails to fully reveal the diversity and complexity that may exist between different groups of medical students [4,5].

In light of these challenges, it becomes imperative to understand the extent and nature of PHSS among medical students. This understanding is crucial for developing targeted interventions that can effectively address and mitigate the PHSS. The primary objective of this research is threefold: Firstly, to investigate the latent profiles of PHSS among medical students in Hainan Province, thereby providing a nuanced understanding of this stigma. Secondly, to analyze the psycho-social factors that influence these latent profiles of PHSS. This involves examining the variables that contribute to or mitigate the PHSS. Finally, the study aims to determine a quantifiable threshold for PHSS among medical students using receiver operating characteristic (ROC) analysis. This threshold will aid in identifying students at risk of experiencing heightened levels of stigma. By accomplishing these objectives, the study seeks to offer a scientific basis for developing personalized mental health interventions tailored to the needs of medical students. It aims to promote more precise and in-depth research on PHSS, ultimately facilitating better mental health support for this specific academic population.

## 2. Methods

### 2.1 Participants and sampling

This study was conducted in Hainan Province, China, from February to July 2023. Hainan Province is located at the southernmost tip of China and includes territories such as Hainan Island, the Paracel Islands, the Spratly Islands, and the Zhongsha Islands. Within this region, three prominent medical institutions are located: Hainan Medical University, Hainan Health Management College, and Hainan Health Vocational College. The sample size for this study was estimated using the formula N =  $\frac{t^{2}p\left(1-p\right)}{e^{2}}$, with a 99% confidence level corresponding to t = 2.56; Drawing from domestic Chinese study, the rate of seeking professional psychological help is approximately 4%, which corresponds to P = 4%; The allowable sampling error is ≤  1%, meaning e = 1%. After accounting for a 10% inefficiency rate in the questionnaires, a sample size of approximately 2797 individuals is required.

### 2.2 Pilot and formal survey

#### 2.2.1 Pilot survey.

The pilot survey for this research was carried out among 60 medical students at Hainan Medical University. After conducting a pilot test with a small sample of medical students, several corrections were made to the questionnaire, including rephrasing certain items for clarity, adjusting questions for cultural relevance, and improving the format and layout for readability.

At the same time, the outcomes of the pre-survey indicated that the survey questionnaire utilized in this study satisfied the required reliability and validity standards, thus no alterations will be made to its content. Consequently, we will incorporate the 60 pre-survey questionnaires into the overall collection for subsequent processing and analysis. To ensure the reliability of our findings, a sensitivity analysis was conducted to determine whether the inclusion of the pilot sample influenced the final results. The analysis revealed no significant differences in the outcomes with or without the pilot sample, confirming that the inclusion of the pilot sample did not affect the validity of the final analyses.

#### 2.2.2 Formal survey.

This study employs a cross-sectional survey design, utilizing stratified cluster random sampling in conjunction with questionnaire surveys. Given the difficulty of contacting the fifth year of undergraduate studies due to their internship requirements in hospitals and other institutions, we opted to sample students from five different grade levels: freshmen, sophomores, juniors, seniors, and graduate students. This meticulous stratification approach enables us to more precisely analyze PHSS differences among students of various grades. Within each grade stratum, we randomly selected five majors, and ultimately, randomly sampled students from one class in each major to serve as the research sample.

Since there was overlap in the majors chosen by each college, 11 distinct majors were ultimately included, such as preventive medicine, medical laboratory technology, clinical medicine, nursing, anesthesia, imaging, and drug management. Subsequently, a representative class from each academic year in the selected disciplines was randomly chosen. In our study, we only considered the grade level of medical students, given that graduate education is primarily centered on their tutors rather than classes, and due to the limited number of graduate students willing to participate in this study, we treated all graduate students in this study as a single grade level class for recruitment and analysis purposes.

Subsequently, Subsequently, we included the students from the selected classes based on the following criteria: (1) being a registered student in one of the three selected institutions, (2) aged 18 years or older, (3) willing to participate in the survey. The recruitment process included a clear explanation of the study’s objectives and procedures, ensuring that participation was voluntary and based on informed consent. Through classroom meetings, well-trained researchers used Mandarin (the official language of China) to provide a detailed introduction to the survey content for the students, ensuring that participants could fully understand the research procedures, objectives, and their rights, including the right to withdraw from the study at any time without any adverse consequences. Since all participants are adults, we do not need to obtain consent from parents or guardians. After the participants completed the questionnaire, we immediately collected the questionnaires, using this as an indication of their consent to participate in the research.

Following the formal survey, questionnaires were completed by 3650 medical students from three institutions. Two team members verified the data’s accuracy and entered it into EpiData 4.6.0.0 software. Of the 3650 questionnaires collected, 197 (5.40%) were excluded due to logical errors or missing information. Logical errors included: respondents may provide conflicting answers on the same scale; answers to the questionnaire do not match the predetermined conditions or scope; obvious errors in the sequence of questionnaire answers, such as consecutive choices of the same or extreme values, may indicate that the respondents did not fill out the questionnaire carefully, but randomly or mechanically selected the answers. Ultimately, 3453 self-reported questionnaires were left for our analysis, with a response rate of 94.60%.

### 2.3 Measures

#### 2.3.1 Ethics statement.

In accordance with the Declaration of Helsinki, the study adhered to ethical principles. Ethical approval for this study was provided by the Human Research Ethics Committee, Hainan Medical University, Haikou, China (HYLL-2022-210). Each participant was voluntary andidentity information of all participants was kept strictly confidential.

#### 2.3.2 Demographic characteristics.

In order to collect relevant information for the research, we designed a demographic questionnaire to collect key demographic information. The variables included in the questionnaire were selected based on extensive literature review and previous research results to ensure that they are closely related to the research objectives. The main information we collected includes key variables such as gender, academic year, whether only-children or not, self-assessed perceived stress, self-assessed academic competition, and family socioeconomic status.

Self-assessed Perceived Stress was evaluated using a single item, ‘How would you rate your overall stress level during the past month?’, ranging from ‘no stress at all’ to ‘very high stress’. Self-assessed Academic Competition was assessed with the item ‘How would you rate the level of academic competition you experience at university?’, ranging from “no competition” to “very intense competition”. Both variables are based on the Likert 5-point scale, with scores from 1 (none) to 5 (very).

#### 2.3.3 PHSS.

The Self-Stigma of Seeking Help Scale (SSOSH) is a scale developed by Vogel et al [7]. In this study, we utilized the SSOSH Chinese version, which was revised by Hao Zhihong and Liang Baoyong in 2011 based on the original scale designed by Vogel et al. for seeking help and the stigma associated with receiving psychological help developed by Komiya et al [8]. The revised Chinese version of the scale is more suitable for measuring PHSS among Chinese university students in various aspects. The scale includes 10 questions, and participants we [9] asked to rate each item on a 5-point Likert scale to reflect their actual opinions, ranging from 1 (“strongly disagree”) to 5 (“strongly agree”), with a total score range of 10-50 points. The higher the score on this scale, the greater the likelihood of experiencing PHSS [9]. In the present study, the Cronbach’s alpha coefficient for the SSOSH scale was determined to be 0.757, indicating a satisfactory level of internal consistency.

#### 2.3.4 Family socioeconomic status.

The Family Socioeconomic Status Questionnaire is an instrument designed to assess the socioeconomic status of college students’ families. The scale, developed by Shi Baoguo and Shen Jiliang [10], was used to categorize parental occupation into five levels, ranging from unemployed or unskilled workers to senior professionals and managers. The educational level of parents is classified into six grades, from primary school to graduate studies, with points assigned accordingly. Higher scores reflect a better economic situation. The Cronbach alpha coefficient of the scale was 0.86.

#### 2.3.5 Professional help-seeking attitude.

The Attitudes Toward Seeking Professional Psychological Help-Short Form (ATSPPH-SF) was utilized to measure the psychological help-seeking attitude of college students. This scale, revised by Fischer and Farina in 1995, comprises 10 items spanning two dimensions: openness to seeking treatment for emotional problems and the value and need in seeking treatment. Ratings are based on a 4-point Likert scale, ranging from “disagree” (0 points) to “agree” (3 points) [11]. Higher scores indicate more positive attitudes towards seeking psychological assistance. This study adopted the Chinese version revised by Kong Xueyan et al., which demonstrated strong internal consistency reliability in its validation testing [12,13]. The Cronbach alpha coefficient in this study is 0.713, suggesting that the data reliability is within an acceptable range.

#### 2.3.6 Mental health level.

The General Health Questionnaire-20 (GHQ-20), developed by Goldberg and revised for Chinese populations by Dr. Li Hong et al., is a widely used tool for assessing mental health in non-clinical settings [14]. This 20-item questionnaire focuses on common mental health issues such as somatic symptoms, anxiety, social dysfunction, and severe depression. Designed for self-administration, it asks respondents to compare their current state to their usual functioning on a four-point Likert scale. The GHQ-20’s binary scoring system (0–1) leads to a total score range from 0 to 20, where higher scores indicate a greater likelihood of a mental health disorder. Recommended for its brevity and reliability, the GHQ-20 is particularly effective in diverse cultural contexts, including China [15]. In this study, it demonstrated a high Cronbach alpha coefficient of 0.973.

#### 2.3.7 Self-efficacy.

The General Self-Efficacy Scale (GSES) is used to measure the self-efficacy of college students. The Chinese version, developed by Prof. Ralf Schwarzer and translated and revised by Wang Caikang et al. (2001) [16], was used. The GSES consists of 10 items, each rated on a 4-level scale. Higher scores correlate with stronger self-efficacy. The Cronbach alpha coefficient of the scale was 0.87.

#### 2.3.8 Family function.

The Adaptation, Partnership, Growth, Affection, Resolve (APGAR) scale evaluates the family function of college students. Designed by Smilkstein in 1978 in the United States, the scale includes five questions, each representing a dimension of family function [17]. Responses are scored from 0 to 2, with higher scores indicating better family functional status [18]. In the context of Chinese populations, the APGAR scale has been proven to have excellent reliability and validity [19], and the reliability coefficient of the scale was 0.78 in this study.

### 2.4 Statistical analyses

The primary hypothesis tested with Latent Profile Analysis (LPA) models is that there are distinct latent profiles of PHSS among medical students. Using Mplus 8.3 to estimate LPA models, which are designed to identify individuals based on their characteristics across a set of variables and categorize them into different latent profiles [20]. This method is based on mixture model theory, assuming that the data consists of multiple distinct latent profiles, and employs Full Information Maximum Likelihood (FIML) estimation and robust standard errors to calculate the specific parameter values for each class [21]. The LPA process was conducted iteratively, fitting models ranging from two to five classes. This involved a comparative analysis of models using both relative and inferential fit indices, such as the Akaike Information Criterion (AIC), the Bayesian Information Criterion (BIC), sample size-adjusted BIC (aBIC), the Vuong-Lo-Mendell-Rubin likelihood ratio test (LMR), the bootstrapped likelihood ratio tests (BLRT) and entropy [22]. Smaller values for BIC, adjusted BIC and AIC indicated a better model fit. A significant p-value for VLMR LRT indicates the (K-1)-class model has to be rejected in favor of a model with at least K-classes. In addition to the fit indices, we explored the identified class compositions to ensure the subgroups are meaningful. Further, the model entropy values closer to one (range: 0–1) was used as an indicator of a good classification [20,23]. In addition, the selected model should ensure a uniform distribution of sample sizes across all profiles, with each category having a sample size greater than 5% [21].

As a follow-up to LPA, we will use the profiles determined by LPA as the dependent variable to explore the factors that influence the assignment to different profiles. This step will help us to better understand the characteristics of each latent profile and to identify key variables that may influence the classification into these groups. We conducted subsequent analyses using SPSS 24.0, prior to conducting parametric tests, normality of continuous variables was assessed using the Kolmogorov-Smirnov test. Counting data were represented as frequency and percentage, and group comparisons were made using the chi-square test. Subsequently employing the chi-square partitioning method for post-hoc comparisons. For rank data, we employed the rank sum test. Measurement data adhering to a normal distribution were expressed as mean ±  standard deviation, with inter-group comparisons conducted via the two independent samples t-test. Conversely, data not conforming to normal distribution were presented through median and quartile, and conducting multiple group comparisons using the Kruskal-Wallis Test, followed by Bonferroni method for post hoc comparison. In addition, Spearman rank correlation analysis was used to evaluate the correlation between variables.

Furthermore, logistic regression analysis tested the hypothesis that lower professional help seeking attitudes, mental health levels, self-efficacy, and family functioning predicted the likelihood of being classified as higher PHSS characteristics (moderate and high stigmatization). Prior to conducting logistic regression analysis, we calculated the variance inflation factor to examine the assumptions of logistic regression, including the absence of multicollinearity, linearity of the log-likelihood function, and the presence of influential outliers. No violations of assumptions were detected, thus no adjustments to the logistic regression model were necessary.

Moreover, the Receiver Operating Characteristic (ROC) analysis played a crucial role in identifying the optimal cut-off value for The Self-Stigma of Seeking Help Scale (SSOSH). This analysis utilized the area under the curve (AUC), along with sensitivity and specificity, to evaluate the performance of the classifiers. The Youden index value was particularly instrumental in determining the optimal cut-off value [24]. The test level was set at α =  0.05, maintaining statistical rigor throughout our analysis.

## 3. Results

### 3.1 Demographic characteristics and questionnaire metrics

As shown in Table 1, the demographic data of 3,453 medical students in Hainan Province was analyzed. Females (53.8%, 1859) outnumbered males (46.2%, 1594). Juniors had the highest proportion (37.9%, 1308) among academic years, followed by freshmen (25.1%, 865), sophomores (21%, 725), seniors (14.5%, 502), and graduates (1.5%, 50). Most (69.1%, 2385) were not only children. For self-assessed stress, 47.8% (1650) had moderate stress. In academic competition stress, 43.2% (1492) had moderate and 33.2% (1145) had high stress.

**Table 1:** Demographic descriptions of 3453 medical students in Hainan province.

Variables		N(3453)	Percentage (%)
**Enumeration Variables**			
Sex			
	Male	1594	46.2
	Female	1859	53.8
Academic Year			
	Freshman	865	25.1
	Sophomore	725	21
	Junior	1308	37.9
	Senior	502	14.5
	Graduate	50	1.5
Only Child			
	Yes	1068	30.9
	No	2385	69.1
Self - assessed Perceived Stress			
	None	192	5.6
	Mild	614	17.8
	Moderate	1650	47.8
	High	772	22.4
	Very High	219	6.3
Self - assessed Academic Competition			
	None	82	2.4
	Mild	387	11.2
	Moderate	1492	43.2
	High	1145	33.2
	Very High	341	10
**Continuous Variables**			**P50(P25,P75)**
PHSS			22 (18,27)
Socio - economic Status			11 (8,14)
Mental Health Level			6 (3,9)
Professional Help - seeking Attitude			17 (15,20)
Self - efficacy			24 (20,29)
Family Function			7 (5,10)

For variables not conforming to a normal distribution, medians and interquartile ranges were reported. Participant scores were as follows: PHSS: 22 (18,27), socioeconomic status: 11 (8,14), Mental health level: 6 (3,9), Professional help-seeking attitude: 17 (15,20), Self-efficacy: 24 (20,29), and family function: 7 (5,10).

### 3.2 LPA models of PHSS among medical students and naming of latent profiles

An exploratory latent profile analysis (LPA) was conducted using the 10 items of the SSOSH to understand PHSS among medical students, resulting in five models with 1 to 5 profiles as shown in Table 1. The AIC, BIC and aBIC decreased with the increase of profile number. The LMR and BLRT test were all statistically significant. The entropies of all profiles were above 0.9. Based on the principle of selecting potential profile models, we ultimately chose. the three-profile model for its balanced distribution and robust statistical support. This decision aligns with the recommendations of several previous studies on using LPA to identify latent profiles based on fit indices and statistical criteria [21,22]. Ultimately, the three-profile model was chosen for its balanced distribution and robust statistical support across these indices (Table 2).

**Table 2:** Potential profile model fitting index of help-seeking stigma among medical students (n = 3453).

Model	AIC	BIC	aBIC	Entropy	LMR(P)	BLRT(P)	Class probability (%)
1	92087.94	92210.88	92147.33	–	–	–	1
2	80087.56	80278.12	80179.61	0.92	<0.00^**^	<0.00^**^	0.68/0.32
3	74097.71	74355.88	74222.43	0.94	<0.00^**^	<0.00^**^	0.23/0.53/0.24
4	72125.24	72451.03	72282.6	0.95	<0.00^**^	<0.00^**^	0.50/0.22/0.24/0.04
5	66023.46	66416.86	66213.51	0.98	<0.00^**^	<0.00^**^	0.18/0.12/0.46/0.19/0.05

**Note:**

**P ≤ 0.001.

**Fig 1:**
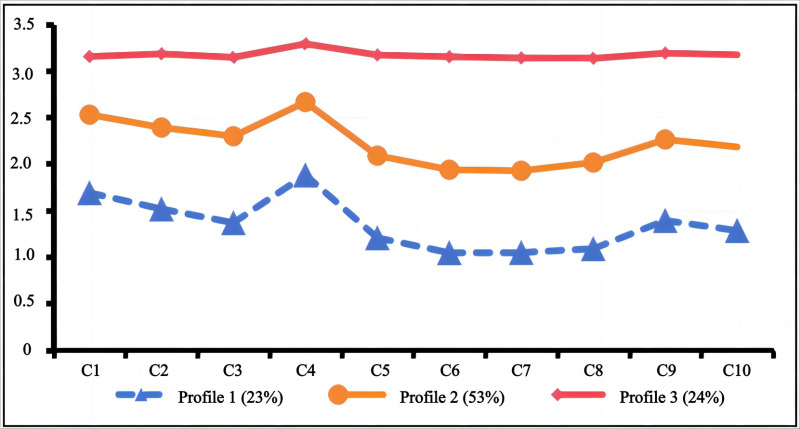
Probability of 10 - item scores for 3 stigma clasXZses in med students.

In this model, Profile 1 (n = 802, 23.0%) showed the lowest PHSS scores, indicating minimal stigma towards professional psychological help-seeking, and was labeled “low-level stigma.” Profile 2 (n = 1836, 53.0%), with moderate scores across the scale, was labeled “moderate-level stigma.” Profile 3 (n = 815, 24.0%), scoring the highest, indicated the most significant level of stigma and was labeled “high-level stigma.” These profiles provide insights into the various degrees of PHSS among medical students. The scoring probabilities of 10 items in the three categories of seeking help for medical students are shown in Fig 1.

### 3.3 Analysis of factors influencing PHSS profiles in medical students

Univariate and multiple logistic regression analyses were conducted to investigate the factors influencing PHSS profiles among medical students. The univariate analysis indicated statistically significant differences in the PHSS profiles of medical students across various demographic and psychosocial variables, including gender, grade, family socioeconomic status, professional help-seeking attitude, mental health level, self-efficacy, and family function (all P < 0.05) (Table 3).

**Table 3:** Univariate analysis of different latent profile [n = 3453, n (%), Pairwise Comparisons ($X^{2}$ or *H/P*)].

Factors	Profile 1	Profile 2	Profile 3	$X^{2}$ /*H*	*P*
	n = 802	n = 1836	n = 815		
**Gender**				79.75	<0.00^**^
Males	302(37.66)	812(44.23)	480(58.90)		
Females	494(62.34)	1010(55.77)	329(41.10)		
Profile 1 - Profile 2	10.86/0.00^**^	Profile 1 - Profile 3	74.11/0.00^**^	Profile 2 - Profile 3	47.48/0.00^**^
**Academic year**				40.75	<0.00^**^
Freshmen	224(27.93)	428(23.31)	213(26.13)		
Sophomores	169(21.07)	400(21.79)	156(19.14)		
Juniors	283(35.29)	718(39.11)	307(37.67)		
Seniors	120(14.96)	270(53.71)	112(13.7)		
Graduate students	6(0.37)	20(0.98)	24(1.96)		
Profile 1 - Profile 2					
Freshmen- Sophomores	2.824/0.09	Freshmen- Juniors	6.538/0.01^*^		
Freshmen- Seniors	1.303/0.25	Freshmen- Graduate students	1.384/0.24		
Sophomores- Juniors	0.362/0.55	Sophomores- Seniors	0.125/0.72		
Sophomores- Graduate students	0.526/0.47	Juniors-Seniors	0.851/0.36		
Juniors-Graduate students	0.338/0.56	Seniors-Graduate students	0.683/0.41		
Profile 1 - Profile 3					
Freshmen- Sophomores	0.05/0.82	Freshmen- Juniors	1.02/0.31		
Freshmen- Seniors	0.02/0.89	Freshmen-Graduate students	10.90/0.00^**^		
Sophomores- Juniors	1.36/0.24	Sophomores- Seniors	0.004/0.95		
Sophomores- Graduate students	11.25/0.0^**^	Juniors-Seniors	0.94/0.33		
Juniors-Graduate students	8.97/0.00^**^	Seniors-Graduate students	10.71/0.00^**^		
Profile 2 - Profile 3					
Freshmen- Sophomores	3.67/0.06	Freshmen- Juniors	1.91/0.17		
Freshmen- Seniors	1.65/0.20	Freshmen-Graduate students	8.32/0.00^**^		
Sophomores- Juniors	0.62/0.43	Sophomores- Seniors	0.18/0.67		
Sophomores- Graduate students	13.62/0.00^**^	Juniors-Seniors	0.05/0.82		
Juniors-Graduate students	11.94/0.00^**^	Seniors-Graduate students	11.55/0.00^**^		
**whether only-children or not**				6.86	0.14
Only-children	265(33.04)	544(29.63)	259(31.78)		
Non-only child	530(66.08)	1280(69.72)	545(66.87)		
**Self-assessed Perceived Stress**				-0.03	0.29
Absent	50(6.23)	80(4.36)	62(7.61)		
Mild	155(19.33)	301(16.39)	158(19.39)		
Moderate	342(42.64)	947(51.58)	361(44.29)		
High	198(24.69)	410(22.33)	164(20.12)		
Very High	56(6.98)	94(5.12)	69(8.47)		
**Self-assessed Academic Competition**				0.03	0.19
Absent	17(2.12)	33(1.80)	32(3.93)		
Mild	101(12.59)	203(11.06)	83(10.18)		
Moderate	340(42.39)	821(44.72)	331(40.61)		
High	268(33.42)	627(34.15)	250(30.67)		
Very High	75(9.35)	150(8.17)	116(14.23)		
**Family Socioeconomic Status** ^**1**^	12(9,15)	11(8,14)	11(8,15)	7.47	0.02^*^
Profile 1 - Profile 2	119.25/0.00^**^	Profile 2 - Profile 3	-39.07/0.35	Profile 1 - Profile 3	80.18/0.11
**Professional Help-seeking Attitude** ^**1**^	20(17,22)	18(15,20)	15(15,16)	590.9	<0.00^**^
Profile 1 - Profile 2	442.56/0.00^**^	Profile 2 - Profile 3	733.82/0.00^**^	Profile 1 - Profile 3	1176.38/0.00^**^
**Mental health level** ^**1**^	4(3,7)	5(3,8)	8(5,11)	250.12	<0.00^**^
Profile 1 - Profile 2	-291.95/0.00^**^	Profile 2 - Profile 3	-474.55//0.00^**^	Profile 1 - Profile 3	-766.50//0.00^**^
**Self-Efficacy** ^**1**^	26(22,30)	24(20,28)	24(20,29)	79.42	<0.00^**^
Profile 1 - Profile 2	385.074/0.00^**^	Profile 2 - Profile 3	-100.446/0.16	Profile 1 - Profile 3	284.628/0.00^**^
**Family Function** ^**1**^	8(5,10)	7(5,9)	5(5,8)	159.78	<0.00^**^
Profile 1 - Profile 2	264.16/0.00^**^	Profile 2 - Profile 3	343.66/0.00^**^	Profile 1 - Profile 3	607.82/0.00^**^

**Note: “1” represents an abnormal distribution variables, which are described by P50 (P25, p75), and the test statistic is H value; mental health level is a reverse score,**

* : P ≤  0.05,

** : P ≤  0.001.

Further, multiple logistic regression analysis was performed, with “low-level stigma” (Profile 1) as the reference group. Significant indicators from the univariate analysis were included as independent variables, with the PHSS score as the dependent variable. This analysis employed a disordered multiple logistic regression approach. Results indicated that factors such as professional help-seeking attitude, mental health level, self-efficacy, and family function significantly impacted the classification into different latent profiles of PHSS among medical students (all p < 0.05) [25] (Table 4).

**Table 4:** Multiple logistic regression analysis(n = 3453).

Factors	Profile 2 (moderate-level)				Profile 3 (high-level)			
	*b*	*Wald* $x^{2}$	*P*	*OR(95%CI)*	*b*	*Wald* $x^{2}$	*P*	*OR(95%CI)*
**Gender**	-0.01	0.05	0.83	0.99(0.98 ~ 1.01)	-0.02	0.07	0.79	0.99(0.98 ~ 1.01)
**Academic year**	0.03	0.68	0.41	1.02(0.96 ~ 1.09)	-0.01	0.24	0.63	0.98(0.94 ~ 1.03)
**Professional Help-seeking Attitude**	-0.14	108.46	<0.00^*^	0.87(0.84 ~ 0.89)	-0.37	376.65	<0.00^**^	0.67(0.66 ~ 0.71)
**Mental health level**	0.05	10.62	0.00^**^	1.05(1.02 ~ 1.08)	0.16	76.44	<0.00^**^	1.17(1.13 ~ 1.21)
**Self-Efficacy**	-0.01	4.38	0.04^*^	0.98(0.97 ~ 0.99)	0.02	3.12	0.08	1.01(0.99 ~ 1.03)
**Family Function**	-0.04	4.26	0.04^*^	0.95(0.92 ~ 0.99)	-0.11	19.27	<0.00^**^	0.89(0.85 ~ 0.94)
**Family Socioeconomic Status**	-0.05	0.66	0.42	1.00(0.99 ~ 1.01)	-0.05	1.23	0.27	1.01(0.99 ~ 1.03)

**Note: take the Profile1 “low-level stigma” as the reference class;**

*  Indicates P ≤  0.05,

**  indicates P ≤  0.001.

### 3.4 Receiver operating characteristic (ROC) analysis

**Fig 2:**
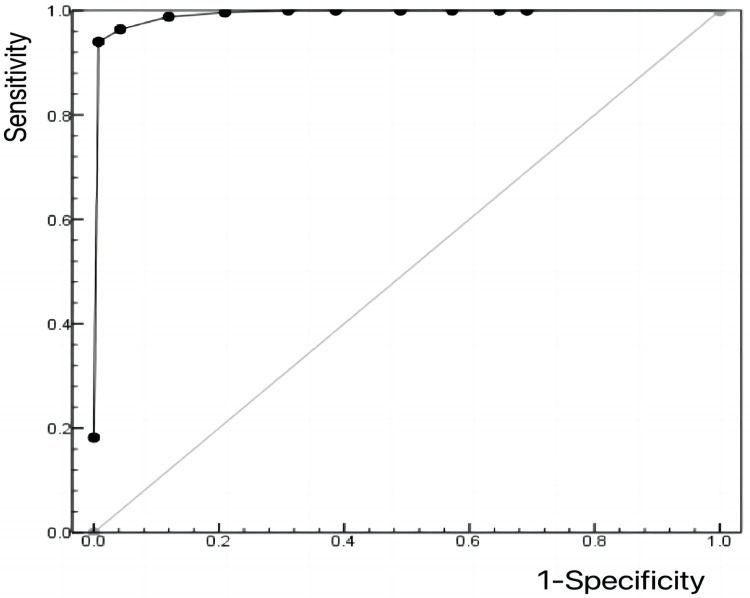
ROC Curve of SSOSH Total Score for Predicting PHSS.

Order to determine the optimal cutoff value for screening medical students’ perceived stigma using the Self-Stigma of Seeking Help Scale (SSOSH), participants in the “low-level stigma”(Profile 1) were defined as “non-cases” in the latent profile analysis, while participants in the “moderate-level stigma” (Profile 2) and “high-level stigma” (Profile 1) were defined as “cases”. The ROC curve was then plotted for the SSOSH total score using the binary outcomes, with an AUC value of 99.4% (p <  0.001), indicating a good predictive capacity for PHSS (see Fig 2). The diagnostic criteria and indices are illustrated in Table 5. The optimal cutoff value was ≥ 19, where the sensitivity, specificity and Youden index value were 0.940, 0.993, and 0.933, respectively.

**Table 5:** ROC curve graph standard values and coordinates.

Criterion	Sensitivity	Specificity	Yoden index
>10	1.000	0.308	0.308
>16	0.996	0.791	0.787
>19	0.940	0.993	0.933
>24	0.462	1.000	0.462
>30	0.127	1.000	0.127

## 4. Discussion

### 4.1 Variability in PHSS among medical students in Hainan province

This study revealed that the median score of PHSS among medical students in Hainan Province was 22 points, aligning closely with findings from domestic studies on college students’ stigma scores (23.01 ± 4.82, 21.90 ± 6.82) [21,26]. The application of latent profile analysis (LPA) was instrumental in identifying individuals with varying levels of PHSS. By employing LPA, this research successfully categorized PHSS scores into three distinct profiles. Notably, the analysis revealed that nearly 80% of medical students in Hainan Province exhibited medium-level or higher stigma for seeking professional psychological help. This figure is significantly higher compared to international studies, where the proportion of medical students with PHSS ranges from 20.10% to 62.00% [4,5,27–29].The higher prevalence of PHSS observed in Hainan Province underscores the necessity for heightened attention and tailored intervention strategies. It is imperative to address this stigma, as it can impede medical students from seeking the psychological support they might need. The variability in stigma levels also suggests that interventions should be customized to effectively cater to different stigma profiles, thereby enhancing the overall mental well-being of medical students.

### 4.2 Influential factors of PHSS profiles in medical students

This study identifies several key factors influencing the PHSS profiles among medical students. Notably, medical students’ attitudes toward professional psychological help are key factors influencing their latent profiles of psychological help-seeking stigma (PHSS). Specifically, more negative attitudes correlate with a higher likelihood of falling into profiles characterized by elevated levels of PHSS. This finding aligns with multiple studies suggesting a link between attitudes toward professional help and PHSS [25]. In the high-pressure environment of medical education, where resilience and professionalism are highly valued [5], students may harbor negative views about seeking help, perceiving it as a sign of weakness. This internalized stigma can make them reluctant to seek assistance. Moreover, the fear of being negatively perceived by peers or faculty can further perpetuate this internalized stigma, increasing their hesitancy to seek help and deepening the stigma they feel [30]. Importantly, this fear of stigma does not necessarily increase their existing stigma but may reinforce and sustain it by preventing them from seeking help that could alleviate their distress.

Additionally, self-efficacy emerged as a significant influencer. Lower self-efficacy is associated with a higher likelihood of falling into high-level PHSS profiles, as students with lower self-confidence may feel ill-equipped to handle mental health issues, thus intensifying their stigma concerns [31,32]. This lack of self-efficacy can lead to feelings of helplessness and isolation, exacerbating fears of societal judgment and bias regarding their mental health [33,34]. Consequently, these students are more prone to experiencing and perceiving a higher level of PHSS. To address this, it is recommended that medical education institutions focus on boosting students’ self-efficacy. Initiatives such as promoting proactive problem-solving and enhancing confidence in handling challenges could reduce the perceived PHSS. Such measures are expected to encourage greater utilization of mental health services among medical students, ultimately fostering better mental health outcomes [35].

Family function also plays a crucial role. Poor family functioning, indicating weaker communication and emotional support within the family, can exacerbate feelings of insecurity and inferiority. Such an environment might increase the likelihood of falling into high-level stigma profiles, as students fear judgment and stigma [35,36]. To mitigate this, enhancing family education and mental health awareness is essential, particularly in families with functional challenges. Implementing family education programs and offering family counseling services can foster healthier interactions and support systems within families. These interventions aim to improve family functioning, thereby creating a more supportive environment for individual psychological growth and reducing the PHSS.

It is significant that medical students with lower mental health levels are more inclined to fall into high-level latent PHSS profiles. Contrary to expectations, these students, who arguably require professional help the most, are often the most reluctant to seek it due to the PHSS [2]. This paradox highlights a critical issue: the stigma effectively prevents those in dire need of mental health support from accessing it. Mental health challenges can lead to self-stigmatization, where students label themselves as “weak” or “abnormal” [29]. Such internalized negative perceptions may deter them from seeking help, as it could be seen as admitting to these perceived flaws [37]. Understanding this dynamic is crucial for developing interventions that not only address mental health issues but also tackle the associated stigma. By doing so, we can ensure that all students, especially those most at risk, receive the necessary support to improve their mental health and overcome barriers to seeking help.

### 4.3 Determining the cut-off value for SSOSH and its implications in medical student mental health

In this study, the cut-off value for the Self-Stigma of Seeking Help Scale (SSOSH) was determined to be 19, established through LPA and Receiver Operating Characteristic (ROC) analysis. This threshold is pivotal for future epidemiological research on PHSS among medical students, offering a critical benchmark for identifying those at risk of high stigma levels. The cut-off value holds considerable practical significance, particularly in the context of clinical practice aimed at promoting mental health among medical students [38,39]. When recommending the screening of PHSS among students, it is crucial to consider ethical issues such as informed consent, confidentiality, and the potential psychological impact on the students being screened. Informed consent should be obtained from all participants, ensuring they are fully aware of the purpose of the screening, how the data will be used, and their right to withdraw at any time. Confidentiality must be strictly maintained to protect students’ privacy. Additionally, support services should be made available to students who may experience distress as a result of the screening process. By addressing these ethical considerations, institutions can proactively address the psychological needs of students, especially those potentially facing higher levels of stigma, thereby contributing to improved mental health outcomes and fostering a supportive environment for their academic and personal growth. By doing so, these institutions can proactively address the psychological needs of students, especially those potentially facing higher levels of stigma, thereby contributing to improved mental health outcomes and fostering a supportive environment for their academic and personal growth.

### 4.4 Limitation

This study has two main limitations. Firstly, a cross-sectional research design was employed, which limits our deep understanding of the changes in PHSS over time. Secondly, the sample was limited to students from three medical colleges in Hainan Province, which may have an impact on the universality of the research results and makes it challenging to generalize to other regions and medical student populations.These limitations need to be considered when interpreting the research findings and identifying future research directions.

## 5. Conclusion

This study by conducting an LPA on the PHSS among Chinese medical students, unveiled significant heterogeneity in this regard among medical students. Nearly 80% of these students exhibited a moderate to high level of PHSS, which undoubtedly warrants immediate attention. Through further analysis, we identified psychological help-seeking attitudes, family functioning, self-efficacy, and mental health levels as factors influencing the latent profiles of PHSS among medical students. In practical terms, this finding not only provides us with a deeper understanding of the potential for high PHSS among medical students but also points us towards potential intervention strategies. Firstly, after identifying these influencing factors, medical colleges and universities can develop more targeted mental health intervention strategies. For instance, students with insufficient family functioning can be provided with family counseling and guidance; for those with a negative attitude towards seeking psychological help, specialized lectures and activities can be implemented to shift their perceptions and alleviate their stigma. Additionally, providing capacity-building training and courses for students with low self-efficacy is also a direction worth considering. Although medical colleges and universities already provide mental health education for students, considering these new findings, it is necessary to further strengthen and improve them. Specifically, greater efforts should be made in mental health outreach work, with the primary goal of reducing the PHSS among medical students and university students, thereby better meeting their mental health needs. Finally, this study also identified the precise critical value of the Self-Stigma of Seeking Help Scale (SSOSH), providing a new tool for screening medical students and even university students who perceive high stigma, and can be used to identify students who need intervention measures.
